# A case of pruritic hyperkeratotic papules on the back

**DOI:** 10.1016/j.jdcr.2023.06.043

**Published:** 2023-07-19

**Authors:** Nedyalko N. Ivanov, Ashley Garvin, Brett Bender, Stephen Olsen

**Affiliations:** a Beaumont Health—Department of Dermatology, Farmington Hills Campus, Farmington Hills, Michigan; b Department of Education, Michigan State University, College of Osteopathic Medicine, East Lansing, Michigan; c Trinity Health - Dermatopathology, Ypsilanti, Michigan

**Keywords:** eruptive pruritic papular porokeratosis, porokeratosis

**Fig 1:**
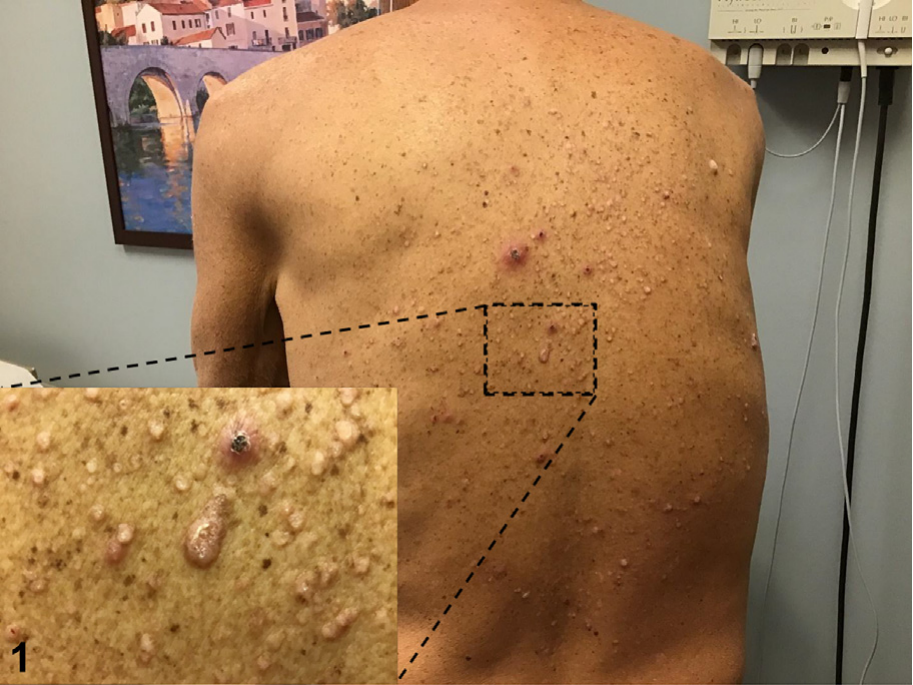

**Fig 2:**
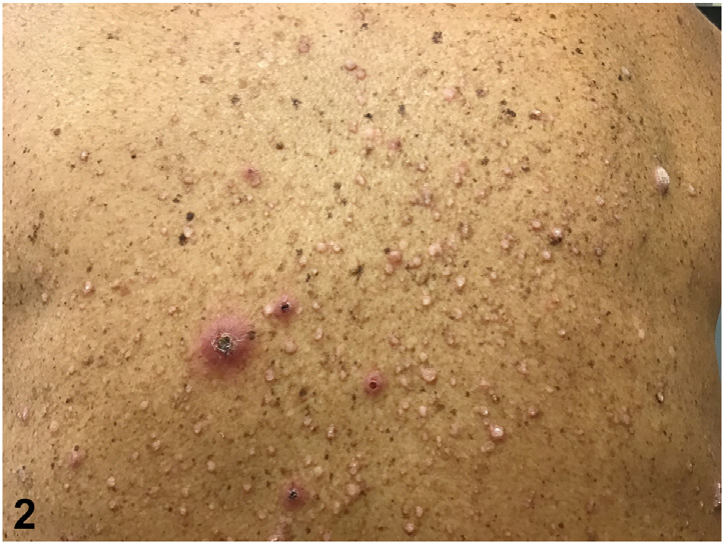

**Fig 3:**
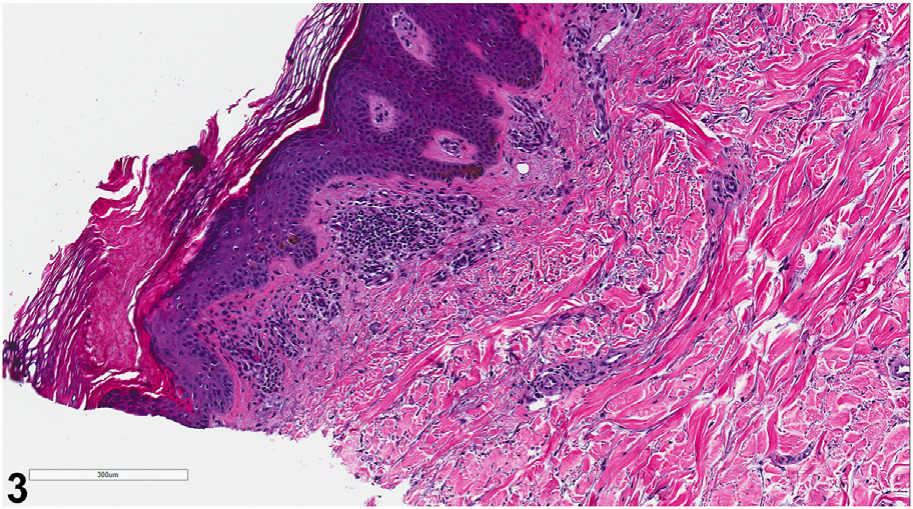

Patient is a 59-year-old man with past medical history significant for hypertension, end-stage renal disease, on dialysis, who presented to the dermatology clinic for intensely itchy lesions, which had erupted over 1 to 2 months. On physical examination, there were scattered erythematous, hyperkeratotic, and excoriated papules with faint collarettes of scale on his back (Figs 1 and 2). Review of systems including fever, chills, fatigue, weight loss, and myalgias were negative. After the patient failed to improve with a trial of topical corticosteroid therapy, a 4 mm punch biopsy was taken from the left lower back for further evaluation (Fig 3).

**Question 1: Which of the following is the most likely diagnosis?**

A: Transient acantholytic dermatosis (Grover’s disease)
B: Eruptive pruritic papular porokeratosis (EPPP)
C: Prurigo nodularis
D: Guttate psoriasis
E: Acquired perforating dermatosis

**Answers:**

A: Transient acantholytic dermatosis (Grover’s disease) - Incorrect. Grover’s Disease classically presents as a sudden onset of pruritus on the back, chest, and upper arms in older men. Lesions are scattered erythematous crusted papules with scant overlying scale that may be eroded. Although Grover’s Disease may be clinically indistinguishable from EPPP, histological findings (Fig 3) are most consistent with the classic findings of porokeratosis (see Question 2 for further discussion).
B: EPPP - Correct. Porokeratosis is a group of keratinization disorders characterized by keratinocyte expansion.^1^ EPPP is a rare subtype of porokeratosis characterized by the sudden onset of sharply demarcated annular plaques with atrophic centers and a thinly elevated keratotic border.^2^ Unlike more common subtypes, EPPP’s involvement is extensive and develops insidiously. Patients present with intensely itchy annular papules, most commonly on the extremities and trunk.^3^ EPPP primarily affects older patients (mean age of onset of 65.8 years old) and is more common in males4. Additionally, EPPP is associated with underlying malignancy in 30% of individuals, most commonly of gastrointestinal and hematological systems.^4^ This correlation may warrant further diagnostic investigation in the appropriate clinical context.^5^
C: Prurigo nodularis - Incorrect. Prurigo nodularis presents as multiple, discrete firm dome-topped nodules which are severely pruritic. These lesions are more common on the extensor surfaces and anterior thighs and slowly enlarge over time.
D: Guttate psoriasis - Incorrect. Guttate psoriasis is a variant of psoriasis that presents as an eruption of many well-demarcated, hypopigmented papules with scale. It commonly presents 2-3 weeks after an upper respiratory tract infection and is more common in children and young adults.
E: Acquired perforating dermatosis - Incorrect. Acquired perforating dermatosis commonly affects patients with chronic renal failure, diabetes mellitus, and, rarely, malignancy. Patients present with pruritic umbilicated hyperkeratotic papules on the trunk and extremities.

**Question 2: Which of the following histopathologic findings is seen with this diagnosis?**

A: Coronoid lamella
B: Acantholysis with dyskeratotic cells
C: Irregular epidermal hyperplasia with compact orthokeratosis and focal parakeratosis
D: Intracorneal collections of neutrophils above mounds of parakeratosis
E: Neutrophilic crust with transepidermal loss of elastic fibers and collagen

**Answers:**

A: Coronoid lamella - Correct. Cornoid lamella is a histologic hallmark of all porokeratosis, indicating disordered migration of epidermal cells.^2^ Inflammatory EPPP shows well-developed cornoid lamellae in the older/deeper layers and poorly developed cornoid lamella in the newer pruritic papules.^2^ This variable morphology helps to differentiate EPPP from other subtypes.^5^ The upper layer may also contain eosinophils and/or lymphoid cells near the vasculature of the upper dermis.^2^ Eosinophilic presence is postulated to be the driving force behind EPPP’s intense pruritus.^2^
B: Acantholysis with dyskeratotic cells - Incorrect. Acantholysis with dyskeratotic cells describes Grover’s disease. The location of these patterns is dependent on which subtype of Grover’s disease is present. For example, in Darier disease, acantholysis is confined to the suprabasal region while dyskeratosis predominantly affects the epidermis.
C: Irregular epidermal hyperplasia with compact orthokeratosis and focal parakeratosis - Incorrect. This histology describes findings that can be seen in prurigo nodules. Eosinophilic infiltration is also commonly seen in the dermal, perivascular and interstitial spaces.
D: Intracorneal collections of neutrophils above mounds of parakeratosis - Incorrect. This histology findings is consistent with guttate psoriasis. Other findings include elongation of the rete ridges.
E: Neutrophilic crust with transepidermal loss of elastic fibers and collagen - Incorrect. This finding can be seen in acquired perforating dermatosis.

**Question 3: Which of the following is considered an effective treatment option for eruptive pruritic papular porokeratosis?**

A: Topical steroids
B: Antihistamines
C: Oral prednisolone
D: Diclofenac gel
E: Cryotherapy, CO_2_ laser, or surgical excision

**Answers:**

A: Topical steroids - Incorrect. Treatment for EPPP is limited and often results in little to no improvement of disease course,^4^ therefore therapy is aimed at symptomatic improvement only. Topical steroids have shown minimal effectiveness at improving symptoms of EPPP.^2^^,^^4^
B: Antihistamines - Incorrect. Antihistamines have shown minimal to no effectiveness at improving pruritus symptoms of EPPP.^2^^,^^4^
C: Oral prednisolone - Incorrect. Oral prednisolone has not been shown to be effective at improving symptoms of EPPP. Alternatively, intralesional steroids may provide symptomatic relief in some patients,^2^ however repeated injections are often required to control symptoms.
D: Diclofenac gel - Incorrect. Diclofenac gel was shown to be useful in some studies at improving symptoms initially, however a rebound eruption occurred after discontinuation, making this option less useful than other options for management of EPPP.
E: Cryotherapy, CO_2_ laser, or surgical excision - Correct. Treatment for EPPP is limited and often results in little to no improvement of disease course,^4^ therefore therapy is aimed at symptomatic improvement only. Cryotherapy, CO2 laser or surgical excision has been reported to provide moderate symptomatic relief for patients in some instances.^3^ Other common interventions include topical urea, vitamin D3, 5-fluorouracil, and systemic retinoids.^3^ On average, lesions persist for six months regardless of treatment and resolve spontaneously in 75% of individuals.^4^
